# The Interaction Between Text Modality and the Learner’s Modality Preference Influences Comprehension and Cognitive Load

**DOI:** 10.3389/fpsyg.2019.02820

**Published:** 2020-01-09

**Authors:** Janina Lehmann, Tina Seufert

**Affiliations:** Department of Learning and Instruction, Ulm University, Ulm, Germany

**Keywords:** modality preference, Learning-Styles Hypothesis, Meshing Hypothesis, text modality, cognitive load, aptitude–treatment interaction

## Abstract

This study investigates the aptitude–treatment interaction between text modality and learners’ modality preference on learning outcomes and cognitive load, which is currently a point of controversy. The Meshing Hypothesis postulates there are better learning outcomes when the modality of a learning environment matches the learner’s preference. However, previous research supporting the Meshing Hypothesis shows methodological issues. Therefore, clear empirical support is needed. We tested 42 learners in a between-subject design: Their preferences were either auditive–ambiguous or visual, and half of each preference group randomly learned either with an auditive or a visual text. As expected, we did not find any main effects, but a significant interaction between the text modality and the learner’s preference for comprehension outcomes, extraneous cognitive load, and germane cognitive load. Specifically, learners with a preference for visual texts benefit from learning with their preferred modality, they showed higher comprehension scores and less extraneous load when learning from a visual text. Auditive–ambiguous learners showed almost equal results with both text modalities. This might be explained by the fact that most texts in everyday life are presented visually, and therefore learners with an auditive preference needed to develop appropriate reading strategies. Thus, our results partly support the Meshing Hypothesis.

## Introduction and Theoretical Background

When deciding which type of learning materials to use for which kind of task, instructional designers have to consider the cognitive affordances of the learning material. However, the line of research on Aptitude–Treatment Interactions (ATI; Snow, 1989) also points out that not only the learning material and its design affect learning outcomes, but also the learners themselves, with their specific learning characteristics. The question now arises as to whether preferences, as a motivationally driven concept, moderate the effects of specific designs of instruction. Many learners prefer to learn in a specific way, which is known as the Learning–Styles Hypothesis (e.g., Cassidy, 2004; Pashler et al., 2008). For example, the modality preference for either auditive or visual information might play a role: Whereas some learners would rather choose to read a text, others prefer to listen to it (Kuerschner et al., 2005). However, the question of whether learners really perform better while learning with their preferred modality (Meshing Hypothesis; e.g., Pashler et al., 2008) still demands clear evidence because most previous research showed methodological issues (Kirschner, 2017). With this study, we aim to close this gap. In order to investigate the question of whether there is an aptitude-treatment-interaction between the learner’s preferences for auditive or visual text as an aptitude and the text modality itself as the treatment factor, it first needs to be considered how auditive and visual information is processed.

### Visual and Auditive Learning Material

Visual and auditive texts are processed differently, and therefore a learner requires different strategies for processing them (e.g., Baddeley, 1986, 2000; Paechter, 1997; Mayer, 2005). A visual text, on the one hand, has the advantage that the presentation is permanent. This enables the learner to control his or her reading pace and to decide on their own whether they need to repeat single paragraphs or sentences, if necessary (Paechter, 1997; Kuerschner et al., 2006). On the other hand, the presentation of an auditive text is transient, meaning that the learner cannot repeat anything besides relying on rehearsal processes (Hitch et al., 1989; Henry, 1991) or on the auditory recency effect (Penney, 1975; see also Rummer et al., 2008), and therefore needs to be very attentive the whole time. Rehearsal is a process which refreshes the decaying information by repetition in the phonological loop (Baddeley, 1986), whereas the auditory recency effect states that there is a memory advantage for the last items in auditory processing. Both effects are less stable compared to simply rereading a visual text. In addition, visual and auditive texts differ in regard to the number of steps needed for processing (Mayer, 2005): Whereas the surface of a visual text (i.e., the characters) needs to be encoded first, processing an auditive text is less arduous, as it already consists of phonemes.

To sum up, visual and auditory texts differ regarding important text characteristics. This leads to the question as to which advantages promote better learning. Empirical research shows different results. There seems to be an interaction between text modality and specific characteristics of the text (e.g., its difficulty and complexity; Penney, 1975), the learner (e.g., reading ability; Paechter, 1997) or the level of learning outcomes (e.g., Rubin et al., 2000).

The most important result for this study is that the presentation of an auditive text seems to be more beneficial with easier texts (Penney, 1975; Paechter, 1997; Rubin et al., 2000). A visual text, on the other hand, should be used if the content is very complex because the permanent presentation format gives the learner the possibility to control the learning progress, for example, by repeating specific phrases which need more attention (Rickheit and Strohner, 1983; Paechter, 1997; Kuerschner et al., 2007). That learners actually make use of this possibility of control can be demonstrated in studies where learners slowed down when reading complex texts or unfamiliar words (e.g., Baker and Brown, 1984). In particular with prosaic texts, comprehension questions can be answered more easily after listening to an auditive version of the text compared to the visual version, while recall questions were answered better after reading the text compared to listening to it (Penney, 1975; Rubin et al., 2000). However, with regards to scientific texts, the learner is confronted with different challenges. In comparison to a prosaic text, more propositions need to be built with a higher element interactivity, which makes coherence building more difficult (Seufert, 2003). In conclusion, adapting the learning process should be more important when learning scientific texts: Learners really needs to have control over the learning process. They need to be able to skip back and forth to build a coherent mental model and to adapt the reading pace to a tempo in which even more difficult phrases can be processed. For scientific texts, this text comprehension strategies would cancel out the reported advantage of auditive texts for comprehension questions, and lead to an advantage of visual over auditive texts for recall and comprehension questions. Thus, we decided to use a scientific text for this study, so that learners are really in need to use text comprehension strategies.

In summary, both text modalities are processed differently: They require different learning strategies and are combined with different advantages. Furthermore, the learner needs to use different strategies for reading and listening. Based on this, it seems plausible that many learners develop a preference for one of these processes, and therefore for one of these two modalities.

### Learner’s Preference for One Modality

Having a preference means that a person prefers to do something in a specific way compared to another way (Kuerschner et al., 2005). Hauck (2003) described two different ways in which such a preference for visual or auditive texts can develop. First of all, successful behavior will be reinforced. Therefore, when learning with one modality is repeatedly successful, an automation of this action will be the consequence. In addition, there is an affective component which influences the learner’s preference. If a learner simply likes the learning material in one modality and assesses this modality as being pleasant, the learner will show a preference for this modality regardless of whether the learning outcome with this modality is successful or not.

Furthermore, the progress of the learner’s reading ability might also play an important role (Paechter, 1997). Especially at a younger age when reading skills are hardly developed, it is much more pleasant to listen to a text than to read one. In general, a modality preference can be described as a stable, but, in principle, adaptable habitual pattern of how to prefer learning, and not as a fixed personality trait (Peterson et al., 2015). However, after reaching the age of adulthood, preferences become more stable and harder to adapt (Kolb, 1984).

The detection of a preference can take place by subjective questioning (metacognitive level; e.g., “In general, do you prefer listening to a text or reading it?”) or by objective decision making (behavioral level; e.g., “You can now decide if you want to learn with a visual or an auditive text. Choose one”). Subjective measuring is only meaningful if the learner is aware of her or his preference, and acts in accordance with the subjectively reported preference. Leutner and Plass (1998) have already proven that it is possible to develop such appropriate questionnaires for visualizer and verbalizer scales. Regarding the preference for an auditive or a visual text, Hauck (2003) assumed that it is also possible to measure them appropriately with a subjective questionnaire (i.e., learners would choose to learn with the modality they report as their preference). He verified his assumption empirically with a medium high significant correlation between his developed subjective questionnaire (Hauck, 2003) and the objective measuring.

Kuerschner et al. (2005) calculated a latent class analysis to identify group characteristics of learners due to their preference for visual or auditive learning material, measured with Hauck’s (2003) questionnaire. In this sample, about 45% preferred visual and 35% preferred auditive texts, with 20% being ambiguous. Learner’s characteristics differed between the two modality preferences. Younger people (age 14–29), men, below-average educated people, students who are still in their vocational training, or workers prefer to listen to a text, whereas middle-aged and older people (age 50+), as well as people with a higher education, predominantly prefer to read a text.

Due to the fact that there are differences between the characteristics of both types of learners, one must consider if one group performs better in a learning task compared to the other. Even though you could assume a disparity due to the different levels of education, a study of Kuerschner et al. (2007) did not find any variations regarding different levels of learning outcomes between the groups. One possible explanation is that the different positive and negative factors for learning counterbalance each other. For this study, data was collected from students, mostly enrolled in psychology courses, which might also influence the composition of the learner’s characteristics in both groups. For example, the participants were mostly young, female adults with generally sophisticated learner characteristics. Therefore, learning outcomes should not differ between different preferences for auditive or visual learning material.

Nevertheless, the interaction between the learner’s preference and text modalities, and therefore the conformity between the preferred modality and the text modality of the learning task, could be an important and influential factor.

### Interaction Between Text Modality and the Learner’s Preference on Learning Outcomes

Interactions between different learner’s characteristics and specific instructional designs, so-called Aptitude–Treatment Interactions [ATI; see Snow (1989) for a general approach], received a lot of attention during the last several years, as “new” media allows one to easily adapt the learning material. Motivational factors, such as the learner’s preference for one modality, may play an important role in aptitudes. Intuitively, one would assume that in general, learners perform better when the learning material considers the learner’s preferences. In keeping with this assumption, the Meshing Hypothesis (Pashler et al., 2008) postulates that learners perform better if the modality of the presented learning material matches the learner’s preference. More specifically, this would mean that auditive learners learn better from an auditive text, and that visual learners learn better from a visual text. Of course, this idea relies on the assumption that different learning preferences really exist (Pashler et al., 2008).

This is based on the idea that learning with the preferred modality leads to increased motivation (Wlodkowski, 2008), whereas learning with the non-preferred modality leads to decreased motivation and frustration (Gilakjani, 2012). Peterson et al. (2015) explained that the interaction between the text modality and the learner’s preference influences the learner’s affect, perception, cognition, and behavior. Altogether, this explains why a learner decides whether to engage her- or himself in the learning process or not (Peterson et al., 2015). In keeping with this argumentation, 93–96% of teachers are convinced that it is important to consider their students’ preferences for different modalities (Dekker et al., 2012).

However, the results of the empirical review of Pashler et al. (2008; see also Cook et al., 2009; Rogowksy et al., 2015) did not show a general advantage of learning with the preferred modality. Pashler et al. (2008) indicated some relevant methodological claims that might explain these inconsistent findings, which should be considered in further research in order to be able to find reliable evidence for the Meshing Hypothesis: The learning styles of the participants have to be measured first. The participants have to learn randomly with their preferred or non-preferred modality, and they all need to complete exactly the same test. All of these demands are clearly considered in this study.

Another question to be considered is whether the differences between learning with the preferred or non-preferred modality are equal for both learning preferences (i.e., whether auditive learners learn equally worse with visual learning material as visual learners do with auditive learning material). In everyday life, most information is presented visually. This means that regardless of the learning type, reading a text is more intensively trained than listening to a text. As a result, learners with an auditive preference are forced to practice their reading skills as well, which will result in the development of reading strategies (Afflerbach et al., 2008). The other way around (i.e., the need to learn an auditive text), is rarely the case. Thus, visual learners do not regularly practice learning with auditive texts. Therefore, we assume that the learning outcomes from learners with a visual preference decrease to a greater extent when learning with an auditive text compared to learners with an auditive preference learning with a visual text.

When talking about learning outcomes, another important variable which needs to be considered might be the specific level of learning outcomes, e.g., recall or comprehension. Recall and comprehension questions differ regarding the level of processing which is needed to answer them correctly, and therefore, they vary in regard to their difficulty. Especially for comprehension tasks, which require to invest more mental effort, it could be important that learners implement all relevant text comprehension strategies (such as repeating single phrases or adapting the reading pace). Thus, learning with the preferred modality might be even more important for completing comprehension tasks.

### Interaction Between Text Modality and the Learner’s Preference on Cognitive Load

Up to this point, we have only spoken about the effects of text modality, the learner’s preference for one modality, and the interaction between both factors on different levels of learning outcomes. In addition, the induced cognitive load is another important measure for this study. The original Cognitive Load Theory (Sweller, 1994; Sweller et al., 1998, 2011; Paas et al., 2003) describes three different types of load: intrinsic (ICL), extraneous (ECL), and germane cognitive load (GCL). ICL is dependent on the inherent complexity of the learning task, and is influenced by element interactivity (Chandler and Sweller, 1991) and prior knowledge (Moreno, 2005). The more elements the learner needs to process simultaneously, the higher the resulting intrinsic load. ECL is caused by the instructional design of the learning task and can therefore be manipulated by its designer. GCL reflects cognitive effort that is germane for learning, and thus, for schema acquisition. This all leads to the question of how the three types of cognitive load are influenced by text modalities, the learner’s modality preference, and the interaction between these two factors. The element interactivity is not influenced by the two modalities, the learner’s preferences, and their interaction. Therefore, there should not be any effects on ICL. As mentioned earlier, it is easier to learn a complex text if the text is presented visually. This is due to the possibilities to control and adapt the learning process that a visual text provides, and which become more important when learning a complex text (Rickheit and Strohner, 1983; Paechter, 1997; Kuerschner et al., 2007). Conversely, auditive texts do not provide this possibility, and therefore learning complex texts auditorily is more difficult. Consequently, the text modality should influence ECL with a higher load while learning with the auditive text. This is also in keeping with results of a study by Leahy and Sweller (2011): They postulated that the modality effect (better learning outcomes when learning a graphic combined with an auditive compared to a visual text) only appears when the content to be learned is simple. For more complex texts, Leahy and Sweller (2011) found reversed results (i.e., that a diagram combined with a visual text was superior to a diagram combined with an auditive text). This was explained by the so-called transient information effect because the time to listen to transient auditive information of complex texts was maybe too long for a direct processing in working memory compared to a visual text (Leahy and Sweller, 2011). Based on this, one would also assume a higher ECL when listening to complex text compared to reading them. However, the learner’s preference should not influence ECL, but instead should influence the interaction between both factors: if the design matches the learner’s preference, then the learner is better acquainted with the specific strategies to learn with this modality, leading to a decreased ECL. In the end, the investment a learner puts into the learning process, thereby causing GCL, should not depend on the text modality or the preference, but on the interaction between both. Learning with the preferred modality should increase GCL because learners like learning with their preferred modality, which should have a stimulating effect.

Even though we acknowledge that there is an ongoing debate regarding the number and nature of the different facets of cognitive loads, our study is based on the original, theoretically sound, and many times empirically investigated Cognitive Load Theory. In contrast to other theories which only differentiate between two different loads, by combining germane and intrinsic load (e.g., Kalyuga, 2011), this theory postulates three different kinds of load, which are mentioned above. Measuring germane and intrinsic load as two different loads is important for our study because we specifically aim to investigate in which way the learner’s effort in schema acquisition (germane load) differs between the preferred and the non-preferred modality.

### Research Questions and Hypotheses

In summary, we wanted to investigate (Q1) the impact of the text modality (auditive or visual) on learning outcomes (recall and comprehension). In this study, we will collect data from psychology students who are predominantly good learners. In order to challenge our participants and to avoid a ceiling effect, we will use a more demanding scientific text with increased complexity. In this case (i.e., with a complex text), being able to regulate the pace or to repeat single phrases should pay off while reading, and also influence ECL.

Based on this argumentation, we hypothesize (H1) a main effect of the *text modality*:

(1a) The visual learning material should lead to better learning outcomes (recall and even more comprehension).
(1b) The visual learning material should lead to a decreased ECL.
(1c) However, ICL and GCL should not be influenced by the text modality.

We also wanted to investigate (Q2) the influence of learner’s preferences on learning outcomes and cognitive load. As described, even though learner’s characteristics differ between both preferences, learning outcomes and cognitive load should not be influenced by them.

Hence, we hypothesize (H2) no main effect of the *learner’s preference*:

(2a) The learner’s preference should not influence learning outcomes (recall and comprehension).
(2b) The learner’s preference should not influence cognitive load (ICL, ECL, GCL).

The most important part of this aptitude-treatment-interaction study is the investigation (Q3) of the interaction between the text modality and the learner’s preference. This is an important and comparably easy-to-implement attempt to foster learning which lacks of empirical proof so far. Controlling all relevant study characteristics, learning with the preferred modality should influence the learner’s invested effort in schema acquisition, and therefore foster learning outcomes.

We hypothesize (H3) an *interaction* between the text modality and the learner’s preference on learning outcomes and cognitive load (ECL and GCL):

(3a) We expect higher learning outcomes (recall and even more comprehension) for all learners who learned with their preferred modality.
(3b) We expect lower ECL scores for all learners who learned with their preferred modality.
(3c) We expect higher GCL scores for all learners who learned with their preferred modality.
(3d) However, the interaction between both factors should not lead to significant results for ICL.

## Materials and Methods

### Pre-study

In a pre-study, we tested the *individual modality preferences* of 223 students of a German university. All participants completed the questionnaire for preferences for auditive versus visual stimuli (PAVS; Hauck, 2003: Kuerschner et al., 2005). The PAVS consists of 12 statements on whether participants would like to learn with auditive or visual learning material on a scale from 1 (*auditive preference*) to 4 (*visual preference*), e.g., “I find it less exhausting to listen to someone compared to reading” or “While reading, I can better focus on details compared to listening” (Hauck, 2003). Learners‘ total PAVS scores are the means of the (reversed) raw scores. Reliability was satisfying, with Cronbach’s α = 0.86 (Hauck, 2003). In his study, Hauck (2003) moreover found that learner’s self-reported preference also matches learner’s behavior when they were actually asked to choose a visual or auditory text. This analysis revealed a satisfying validity of the PAVS: He found a correlation of *r* = 0.54 between a learner’s PAVS score and a learner’s decision to learn an expository text in one specific modality.

To categorize auditive, visual, and ambiguous learners, the results of learners’ PAVS in our pre-study were split at the.33 and.67 percentile (*M*_all_ = 2.84, *SD*_all_ = 0.49; percentile_33_ = 2.60, percentiel_67_ = 3.00). All learners whose scores were in the two extreme groups were invited to take part in our experiment. However, only 42 participants agreed to participate in the main study.

### Main Study

#### Subjects and Design

In the main study, we tested these 42 participants between the ages of 19 and 41 years (*M* = 22.55, *SD* = 4.67), 38 of them were female. Participants were compensated with course credits and signed an informed consent including all relevant information about the study. We implemented an experimental 2 × 2 between-subject design with the experimental factor *modality of the learning material* (auditive or visual). All participants were randomly assigned to one of the two groups. As a second factor, we included the organism variable *learner’s preference* (for either visual or auditory text). Our sample included 19 auditive and 23 visual learners. Taking the group means of the PAVS scale from the pre-study into account, it became clear that the auditive groups must be categorized as an auditive-ambiguous group (*M*_PAVS_ = 2.34, *SD*_PAVS_ = 0.21). Nevertheless, the PAVS scores differed significantly between the auditive and the visual groups [*t*(40) = 11.39, *p* < 0.001]. In total, *n* = 22 students learned with the auditive (*M*_PAVS_ = 2.78, *SD*_PAVS_ = 0.51) and *n* = 20 students learned with the visual learning material (*M*_PAVS_ = 2.81, *SD*_PAVS_ = 0.44). As *dependent variables*, we measured recall and comprehension performance, as well as ICL, ECL, and GCL. As *potential confounding variables*, we considered prior knowledge, age, and gender. A more detailed description of all variables can be found in the following materials section.

This study design fulfills the recently demanded methodological criteria by Kirschner (2017): Participants were randomly assigned to the two experimental groups with half of them receiving the text in the modality matching their learning style and half of them in the modality not matching their learning style. Moreover, all participants took the exact same test.

### Materials

All materials of the main study were paper-and-pencil based, except for the learning material, which was presented on a computer, with additional headphones in the auditive condition.

As *learning material*, we used a scientific text in German about volcanism consisting of 661 words, which was implemented in a longer version by Hauck (2003). The content was presented in 6 chapters, with each chapter being presented on its own slide. Each chapter consisted of 100–150 words. Participants either read the text or heard it with headphones. The record for each chapter in the auditive version lasted for about 2 min. After each chapter in the auditive version, participants had the chance to listen again to the text of the current chapter. The possibility to reread phrases was naturally given in the visual version due to the stability of the text and no time limits on the page. The font color of the visual text was black on a white screen. Words were printed left-justified in Arial, without any additional features, such as highlighting, bold, italics, or different colors. Participants were not allowed to take notes during the learning phase.

The paper-based self-developed pre-test for *prior knowledge* consisted of 10 multiple-choice questions (e.g., “The earth’s surface can be divided into 14 tectonic plates. Correct - Wrong - I don’t know”). *Learning outcomes* were differentiated in recall and comprehension. All answers were compared to predefined solutions and all tests were scored by the same research assistant who was blind to the experimental conditions (for the same scoring method, see e.g., Eitel et al., 2014). To answer *recall* questions, learners had to reproduce the concerning information which was presented in the text. Recall performance was measured with five multiple-choice questions and five open questions (e.g., “What components does the lithosphere have?”). One item for measuring recall was excluded from further analysis because of its negative correlation with the recall scale. Participants could gain 2 points for each correct answer, making a maximum of 18 points in total. Participants received partial points if they wrote parts of the correct answers (e.g., “What components does the lithosphere have?” – 2 points were given, when both components “earth mantle” and “earth crust” were named correctly, 1 point was given when only 1 component was named correctly).

To answer *comprehension* questions, participants needed to link the concepts of the learning material, and thus use their knowledge to extend what was explicitly given in the text. Comprehension was measured with 4 open questions (e.g., “Why does magma rise?”). One item was excluded because of its negative correlation with the comprehension scale. With 2 points for a correct answer, participants could reach a maximum of 6 points. Again, participants received partial points if they wrote parts of the correct answers (e.g., “Why does magma rise?” – 2 points were given, when both aspects “high temperature leads to stone melt” and “magma has a smaller dense” were reported correctly, 1 point was given when only one correct aspect was reported).

In order to measure *Cognitive Load*, participants had to answer the Cognitive Load Questionnaire (Klepsch et al., 2017), consisting of 7 items. Two items measured intrinsic cognitive load (e.g., “For this task many things needed to be kept in mind simultaneously”), three items measured extraneous cognitive load (e.g., “The design of this task was very inconvenient for learning”), and two items measured germane cognitive load (e.g., “For this task, I had to highly engage myself”). Participants determined their agreement with each item on a 7-point Likert-type scale, from 1 = *Not at all* to 7 = *Completely*. In the analysis of the questionnaire (Klepsch et al., 2017), satisfying reliability scores have been reported for all three scales between Cronbach’s α = 0.80 and α = 0.86. In addition, the validity of the scales has been shown by a comprehensive predictive validity test.

A short *demographical questionnaire* was used to assess age, gender, and number of semesters of the participants.

### Procedure

Data collection took place in individual sessions. In the beginning, participants needed to formally agree to the data collection by signing the informed consent. Afterward, all participants completed the demographic questionnaire and the pre-test to measure prior knowledge. Then, participants received the instruction to learn the learning material and that they will have to complete a test afterward. The learning material was visually presented on a laptop or auditorily presented through headphones plugged into a laptop. The maximum time for the learning phase was 20 min. After the learning phase, participants completed the post-test with recall and comprehension tasks, and the Cognitive Load Questionnaire (Klepsch et al., 2017) on paper. The experiment took 40–60 min.

## Results

To test our hypotheses, we set up an ANOVA using SPSS with the two factors modality of the learning material (auditive or visual) and individual modality preference of the participants (auditive or visual). All of our variables were normally distributed (*p*s ≥ 0.10), variance homogeneity (*p*s ≥ 0.15) was given. For descriptive data, see Table 1.

**TABLE 1:** Descriptive data for all variables per condition in percentages.

	**Conditions**							
	**Auditive-Ambigous Preference**				**Visual Preference**			
	**Auditive Text**		**Visual Text**		**Auditive Text**		**Visual Text**	
	***M***	***SD***	***M***	***SD***	***M***	***SD***	***M***	***SD***
Prior Knowledge (%)	29.09	21.19	20.00	16.04	31.81	16.62	26.67	21.88
Recall (%)	54.55	24.70	56.94	18.00	46.97	17.98	62.50	16.60
Comprehension (%)	54.55	23.68	45.83	38.58	39.39	29.13	66.67	20.10
Intrinsic Load (%)	66.23	12.41	60.71	16.64	72.73	13.10	63.10	23.16
Extraneous Load (%)	56.71	15.71	57.74	13.09	65.80	18.30	52.38	15.73
Germane Load (%)	69.16	9.07	72.32	12.19	76.62	9.50	66.37	12.13

### Covariates

We analyzed whether one of the potential confounding variables (age, gender, prior knowledge) has an influence on any of the dependent variables by calculating correlations between the potential confounding variables and the dependent measures. The only significant correlation existed between prior knowledge and extraneous cognitive load (*r* = −0.32, *p* = 0.040). Thus, we included prior knowledge as a covariate in all calculations concerning extraneous cognitive load. Furthermore, we analyzed if the potential confounding variables differed between the groups and found no significant differences.

### Recall

Neither text modality [*F*(1,38) = 2.14, *p* = 0.075, η^2^ = 0.05], nor the preferred modality (*F* < 1, ns), nor the interaction between both factors [*F*(1,38) = 1.15, *p* = 0.29, η^2^ = 0.03], influenced recall performance.

### Comprehension

**FIGURE 1:**
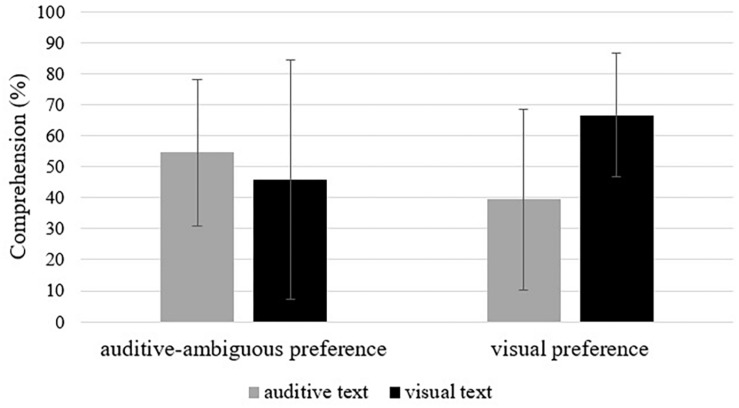
Interaction effect of text modality and modality preference on comprehension (in percent). Error bars are standard deviations.

Neither text modality [*F*(1,38) = 1.16, *p* = 0.14, η^2^ = 0.03], nor the preferred modality (*F* < 1, ns) influenced comprehension. The interaction between the two factors showed significant results [*F*(1,38) = 4.36, *p* = 0.044, η^2^ = 0.10, see Figure 1].

Planned *post hoc* contrasts showed a significant main effect for learning with the preferred modality over the non-preferred modality (*MD* = 35.99, *SE* = 17.24, *p* = 0.022, *d* = 0.69). Calculating subgroup comparisons, this difference is also significant when only visual learners are considered (*MD* = 27.27, *SE* = 11.52, *p* = 0.012, *d* = 1.09), but not when only auditive-ambiguous learners are included (*MD* = 8.71, *SE* = 12.83, *p* = 0.251. *d* = 0.27).

### Intrinsic Cognitive Load

ICL was not influenced by the text modality [*F*(1,38) = 2.02, *p* = 0.082, η^2^ = 0.05], by the preferred modality, or by the interaction between the two factors (*Fs* < 1, ns).

### Extraneous Cognitive Load

Prior knowledge correlates significantly with ECL (*r* = −0.32, *p* = 0.040) and was therefore included as a covariate in the following analysis.

**FIGURE 2:**
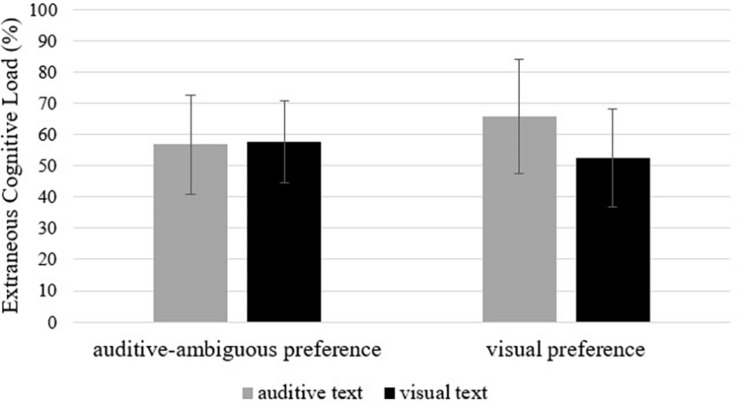
Interaction effect of text modality and modality preference on extraneous cognitive load (in percent). Error bars are standard deviations.

Whereas the preferred modality did not influence ECL (*F* < 1, ns), this load differed significantly between the two modalities of the text (*F* = 5.64, *p* = 0.043, η^2^ = 0.13), with a higher ECL after learning with auditive material. The interaction between the two factors is marginally significant (*F* = 3.51, *p* = 0.083, η^2^ = 0.09, see Figure 2). Even though this *p*-value is only marginally significant, with regard to our rather small number of participants, we decided to calculate all planned *post hoc* contrasts in order to examine our hypothesis. Furthermore, we did not expect all subgroups to differ, only the ones important for our assumptions.

Planned *post hoc* contrasts revealed a main effect: Participants reported a significantly lower ECL while learning with the preferred modality (*MD* = 4.37, *SE* = 2.34, *p* = 0.035, *d* = 0.72). Calculating subgroup comparisons, this difference is also significant for learners with a visual preference (*MD* = 4.20, *SE* = 1.57, *p* = 0.005, *d* = 1.12), but not for learners with an auditive-ambiguous preference (*MD* = 0.15, *SE* = 1.74, *p* = 0.467, *d* = 0.07). The high effect sizes (*d* = 0.72 and *d* = 1.12) confirm that the calculation of the contrasts is right.

### Germane Cognitive Load

**FIGURE 3:**
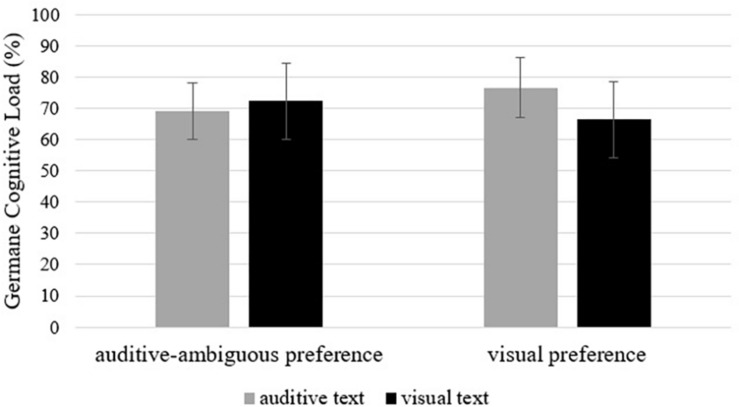
Interaction effect of text modality and modality preference on germane cognitive load (in percent). Error bars are standard deviations.

The groups did not differ in GCL between the two text modalities [*F*(1,38) = 1.17, ns, η^2^ = 0.03], nor in the two preferred modalities (*F* < 1, ns), but we found a significant interaction between both variables [*F*(1,38) = 4.00, *p* = 0.05, η^2^ = 0.10, see Figure 3].

A planned *post hoc* contrast revealed a main effect for GCL: Learners who learned with their non-preferred modality reported higher GCL scores (*MD* = 3.75, *SE* = 1.88, *p* = 0.026, *d* = 0.67). Learners with a visual preference showed a significantly higher GCL while learning the auditive text (*MD* = 2.87, *SE* = 1.26, *p* = 0.014, *d* = 0.94). For learners with an auditive-ambiguous preference, GCL did not differ significantly between the two modalities (*MD* = 0.87, *SE* = 1.38, *p* = 0.265, *d* = 0.29).

## Discussion

### Learning Outcomes and Cognitive Load

The aim of this study was to assess the differences in learning outcomes and cognitive load when learning with the preferred or non-preferred modality, which could be either visual or auditive. Hence, we analyzed the effects of text modality, modality preferences, and especially the interaction between the two factors.

With respect to the overall effect of text modality, we found no influence of text modality on recall and comprehension. We expected an advantage of the visual text because this modality provides a chance for the learner to choose an appropriate reading pace and to repeat challenging paragraphs (Paechter, 1997; Kuerschner et al., 2006). However, the results indicate that learners might not have used this regulative option. This might be due to the fact that our participants did not need to reach a specific level of knowledge in order to get their credit. Therefore, most of our learners probably did not have the aim of really learning the whole text as well as possible, thereby using all the strategies they possess, but instead felt content with simply reading it. This is underpinned by the only medium-high average scores for recall and comprehension. Another explanation would be that the auditive group was also allowed to listen to the auditive text again, and therefore had the chance to repeat complicated paragraphs. Even though they could not jump back and forth for specific phrases, they also had the chance to fill their gaps.

As expected, text modality also did not influence intrinsic or germane load. Intrinsic load is mainly dependent on the element interactivity (Chandler and Sweller, 1991), which stays the same regardless of whether the same text is presented visually or auditorily. Moreover, the learner’s effort into schema acquisition, reflected in germane load (Sweller et al., 1998), did not vary between text modalities. Small differences in load between the groups might be explained by the fact that cognitive load was measured subjectively and participants might have judged the same amount of high load differently.

However, text modality influenced extraneous load, with the auditive version leading to more ECL than the visual text. Hence, it was more exhausting to listen to the text. As described earlier, listening comprehension relies on rehearsal processes (Hitch et al., 1989; Henry, 1991). For a scientific text, such as that used in this study, this seems to be more exhausting than the regulation strategies necessary to learn a visual text (Paechter, 1997; Kuerschner et al., 2007). This is quite intuitive: If the learner has a permanent presentation of a visual text, he or she can simply reread essential paragraphs or jump back and forth in the text to learn it. With a transient presentation of a text that is read out loud, the learner has to remember the wording by him- or herself in order to recall specific parts of the text. This is, of course, more arduous compared to having the text presented in a permanent format. Even though participants had the chance to listen to the auditive text again, they were not able to jump to the specific part, but had to listen to the whole text. But why did the less affording format of the written text not result in better learning outcomes? This might be explained with the results for GCL, which were comparable for the two groups. Thus, despite the fact that the cognitive resources have been freed up for germane activities in the less demanding visual format, overall, learners did not make use of this possibility (the interaction effect on GCL is discussed later on). This again indicates a low commitment of the students to the task.

As expected, the *learner’s modality preference* did not influence recall or comprehension outcomes. This is in line with our hypotheses and with the results of Kuerschner et al. (2007). Furthermore, the learner’s preference does not influence any type of load. Besides element interactivity, prior knowledge is the second factor influencing intrinsic load (Moreno, 2005). However, learners did not differ between the groups regarding prior knowledge leading to equal intrinsic load scores. They all learned with the same instructional material, resulting in equally extraneous load scores. And finally, one half of each group learned with the preferred or non-preferred modality, also resulting in the same motivation to put effort into schema acquisition, and thus in the same amount of germane load.

The *interaction between both independent variables*, which was the focus of this study, did not influence recall performance even though we had assumed an interaction effect. We assumed that learners who had to learn with the non-preferred modality would perform worse when compared to learning with the preferred modality. This should be even more the case for learners with a clear visual preference than for learners in the auditive-ambiguous group. To answer recall questions, a learner only needs to process the surface of the text, which is only slightly demanding (Schnotz and Bannert, 1999). Maybe this is not demanding enough, so that the learner can compensate for learning, even in the non-preferred modality, if the learner is willing to invest more mental effort. Additional support becomes more important for deeper learning processes, such as comprehension (Schnotz and Bannert, 1999). It might be the case that learning with the preferred modality pays off for such advanced learning outcomes where additional support becomes more important.

Giving empirical support to this idea, the interaction between text modality and the learner’s preference significantly influenced comprehension outcomes. As a main effect, participants showed better comprehension performance after learning with the preferred modality. However, this result is mainly due to the results of learners with a visual preference, who profited from the visual text. Learners with an auditive-ambiguous preference answered comprehension questions statistically equally well with both text modalities, even though there were as well descriptively higher comprehension scores when learning with the preferred, auditive modality. Comprehension questions challenge the learner more than recall questions, and demand deeper processing (Schnotz and Bannert, 1999). To be successful, the learner needs to use specific learning strategies appropriately (Meyers and Jones, 1993), whereas we assume that the use of strategies is better trained when learning with the preferred modality.

There are two different explanations as to why visual learners benefited stronger from learning with their preferred modality (*d* = 1.09 for learners with a visual preference vs. *d* = 0.027 for learners with a auditive preference): In our experiment, learners with the highest scores for a visual preference indeed had a clear visual preference, whereas learners with the highest scores for an auditive preference were rather ambiguous. Hence, if you do not have a clear preference, there is not really a preferred or a non-preferred modality. Therefore, there should not be too high differences between learning with visual or auditive texts. The other explanation could be that learners in the auditive-ambiguous group had to become used to reading tasks in everyday life. Most texts are presented visually. Hence, even if one does not like, or is not good at, reading comprehension, he or she would have to practice it almost every day. Afflerbach et al. (2008) assumed that this practicing leads to strategy development. We thus assume that learners with an auditive preference might nevertheless have the opportunity or necessity to develop reading strategies.

Thus, reading tasks will be solved more successfully while developing better strategies (Meyers and Jones, 1993). Hauck (2003) argued that successful behavior strengthens a preference. For learners with an original auditive preference, this could mean that during their education in school, they develop appropriate reading strategies. With increased expertise in using these strategies, we assume that learning outcomes after reading should also improve. The mastery experience could then also lead to a broadened preference toward visual texts (i.e., an auditory-ambiguous preference). However, whether learners with auditive or visual preferences have, in fact, different levels of expertise in reading strategies cannot be inferred from our data, but would have to be analyzed in further studies. The interplay between preferences and intense practice, or even strategy development, is also reflected in the approach of how preferences are developed (Hauck, 2003).

Moreover, learners with a visual preference do not have to practice listening comprehension regularly because it is not the usual format during school education. Therefore, they might not develop appropriate strategies for the processing of auditive texts. Consequently, they suffer more when learning with their non-preferred, and thus less practiced modality. But again, whether learners in fact possess different strategies for visual and auditive texts, depending on their prior experiences and preferences, has to be further clarified.

Although the interaction between the preferred modality and text modality did not influence ICL, it had a strong influence on ECL (Cohen’s *d* = 1.12). In general, learning with the preferred modality leads to less ECL, especially for learners with a visual preference. This supports the assumption that learners with a visual preference possess better learning strategies for their preferred modality, leading to less ECL. Again, we need to point out that we only had a group of auditive-ambiguous learners who knew how to learn with both auditive and visual texts. Therefore, the difference between both modalities did not impact their estimation of ECL. This interaction shows the same pattern as the interaction concerning comprehension.

GCL was also significantly influenced by the interaction between the two factors. Surprisingly, learning with the non-preferred modality led to more GCL than learning with the preferred modality. We thought that learning with the preferred modality would lead to higher motivation (Wlodkowski, 2008), and therefore to an increased effort in schema acquisition of the learner, but, in fact, it was the other way around. However, the increased GCL did not compensate for ECL differences between learners in comprehension. As already mentioned, comprehension questions are demanding. Obviously, learners were not able to compensate for a worse use of strategies by simply trying harder. Cierniak et al. (2008) also argued, based on the results of Tabbers et al. (2000), that a higher GCL does not necessarily lead to better learning outcomes. Furthermore, comprehension questions additionally burden the working memory with more ECL and GCL for learners who learn with the non-preferred modality. The resulting overload is visible in the significantly worse comprehension outcomes when learning with the non-preferred modality. In order to corroborate these assumptions, it would be necessary to measure all three types of load differentiated after each recall and comprehension task. In this study, participants were asked to score all three loads after answering both types of questions.

In conclusion, our results support the Learning-Styles Hypothesis (e.g., Cassidy, 2004; Pashler et al., 2008) because we found different preferences for auditive and visual learning material measured with the PAVS scale. This also supports the idea the learning preferences can be subjectively measured with a questionnaire because we did find significantly different results between the groups. Furthermore, our results partly support the Meshing Hypothesis (Pashler et al., 2008): Visual learners reached better learning outcomes when learning with visual learning material compared to auditive learning material. However, as our sample did not include enough learners with a clear auditive preference, there is still evidence missing to support this part of the Meshing Hypothesis. Moreover, this study fulfils all the criteria demanded by Pashler et al. (2008) when doing research in this area: First, we began by examining the learning style of our respondents. Second, participants randomly learned with their preferred or non-preferred modality. Third, everyone took exactly the same test.

Furthermore, all reported interactions can be classified as crossover interactions, which is required when discussing the Meshing Hypothesis (Pashler et al., 2008). Kirschner (2017) indicated that, so far, there are hardly any studies considering these principles. However, the results of this study are a first indication that the learner’s preferences should be considered while designing a learning environment, and should therefore motivate one to further investigate the Meshing Hypothesis: We need further methodologically tight research, preferably with process data, to better understand what is happening when learning with the preferred or non-preferred modality, and to close the theoretical gap around learner’s preferences. This is especially important with the increasing possibility of learning with computers or comparable media, so that the adaptability of a learning environment can pay off. Leutner (2004) described adaptability as an optimal fit between the learner’s needs and the support provided by the learning environment. This adaptability can only be achieved if the learner’s preferences are measured and considered adequately.

### Limitations, Further Research, and Practical Implications

Even though our study was carefully designed, the results have some limitations. First, the sample of our study is quite selective: We only tested psychology students who were, in general, learners with above-average learning skills and learner characteristics, and above-average grades in high school, as prerequisites to enrolling. Maybe one could find an interaction between text modality and the preferred modality on recall, as well, when the learner is more challenged, either by the complexity of the learning material or by the lack of sufficient learning strategies. Hence, the working memory would be burdened more, and learning with the non-preferred modality would lead to an overload, and a decreased learning performance should be found even on easier recall tasks.

Second, we did not find a group of learners with a clear auditive preference. One possible explanation could be that this preference decreases up until their university education. Students have to complete so many reading tasks during their education that even learners with an auditive preference will adapt specific reading strategies sooner or later. As a result, they find reading more appealing and develop a more ambiguous preference. Further research should also investigate this assumed development to better understand, whether ambiguous learners really adapt their preference or whether they were ambiguous from the beginning. Moreover, it would be interesting to see whether learners with a visual preference could also be trained in listening performance in order to develop a more ambiguous preference. On the other hand, it could be possible to find learners with a clear auditive preference in less educated communities where reading is less practiced, which would be in line with the results of Kuerschner et al. (2007). One further possible option could be to include learners with dyslexia in the sample where an auditive preference could be expected. It would be interesting to repeat this experiment with a group like that, to see if they show differences in learning outcomes and cognitive load between auditive and visual learning material.

And third, the measurement of both cognitive load and comprehension outcomes might be questioned. For cognitive load, we used a subjective measuring tool. Subjective tools may always be biased by incorrect self-perception. But, to our knowledge, up to this point there has been no objective measuring instrument which can differentiate between the three types of load, a feature which was essential for our study. For comprehension, we ended up with only three questions which is rather few compared to the length of the text. Further studies should make sure that they provide a descend number of comprehension questions. Moreover, answers were scored by only one person, whereas one could argue that the scores should have been checked by another, second person.

The last question to be answered is: What do our results mean for everyday learning, for example, the choice for one modality at school or university? The performance of visual and auditive-ambiguous learners never became worse when learning with a visual text, but how it effects learners with a clear auditive preference is still unclear to us. It might be the case that they learn better with an auditive text. Hence, we cannot recommend teaching with only one modality in general. However, in higher educated groups, it seems like there are hardly any clear auditive learners, which supports the approach of teaching with visually presented texts.

Furthermore, people spend increasing amounts of time learning with computers due to the growing relevance of digital media, e-learning, or online study programs. Such learning environments use transient presentation formats, such as auditive texts, but also animations, educational films etc. These formats provide many advantages, such as an easier presentation of procedures. Nevertheless, they are only beneficial if the learner knows how to process them adequately. Thus, we recommend the training of strategies for the processing of transient presentation formats.

## Data Availability Statement

The datasets generated for this study are available on request to the corresponding author.

## Ethics Statement

Ethical review and approval was not required for the study on human participants in accordance with the local legislation and institutional requirements. The patients/participants provided their written informed consent to participate in this study.

## Author Contributions

TS designed and conducted this study. JL wrote this manuscript under supervision of TS.

## Conflict of Interest

The authors declare that the research was conducted in the absence of any commercial or financial relationships that could be construed as a potential conflict of interest.
